# Reliability of ultrasound versus digital vaginal examination in detecting cervical dilatation during labor: a diagnostic test accuracy systematic review

**DOI:** 10.1186/s13089-021-00239-1

**Published:** 2021-08-17

**Authors:** Zaynab Mohaghegh, Shayesteh Jahanfar, Parvin Abedi, Mohamed A. Abd El Aziz

**Affiliations:** 1 Midwifery Department, Ahvaz Jundishapur University of Medical Sciences, Ahvaz, Iran; 2 MPH Program Department of Public Health and Community Medicine, Tufts University School of Medicine, Boston, USA; 3 Department of Midwifery, Menopause Andropause Research Centre, Ahvaz Jundishapur University of Medical Sciences, Ahvaz, Iran; 4 Department of Obstetrics and Gynecology, Benha University Hospital, Benha University, Banha, Egypt

**Keywords:** Ultrasonography, Cervical dilatations, Vaginal exams, Diagnostic test, Labor

## Abstract

**Background:**

This systematic review aimed to investigate the reliability of ultrasound method compared with digital vaginal examinations in detecting cervical dilation.

**Methods:**

We searched Cochrane (CENTRAL), MEDLINE, EMBASE, CINAHL, ISI Web of Science Core Collection, Trip Database, PubMed, DARE and NHS EED, HTA, and PROSPERO. Ten observational studies with a total sample size of 856 were included in the meta-analysis.

**Results:**

The intraclass correlation coefficient (ICC) values ranged between 0.21 and 0.69. The fixed-effect models for the ultrasound test showed an average of ICC (*r* = 0.32 (95% CI 0.26–0.38). Correlation between two methods was poor (*r* = 0.359, 95% CI 0.26–0.44). In nulliparous and multigravida participants the correlation between ultrasound measurements and digital examinations was (*r* = 0.349, 95% CI 0.25–0.43) and ICC (*r* = 0.676, 95% CI 0.419–0.833), respectively.

**Conclusion:**

Trans-perineal ultrasonography seems to be a reliable method for assessing labor progression in multigravida women, but its usage in nulliparous women needs further studies.

## Background

Diagnosing the onset of labor is one of the most critical and complex judgments made by care providers in the delivery room [1]. Measurement of cervical dilatation is considered the most crucial parameter for labor progress during childbirth and the main reason for doing digital vaginal examination (DVE) in women with signs of labor onset [2, 3]. Cervical dilatation is also used to study uterine activity, oxytocin use, and the transition from latent to the active phase of labor and it is an essential element of Bishop Score [4]. Also, cervical dilatation is used to predict the mode of delivery so that people with prolonged labor may have more cesarean sections [5]. Today, the correct measurement of cervical dilation has the utmost importance and help health providers to make a timely and proper decision. Methods used to assess cervical dilatation are classified into three categories: (1) traditional techniques such as mechanical devices, electromagnetic devices, and electronic sensor systems; (2) DVE; (3) and ultrasonic machines [6]. DVE was the most common method for measuring cervical dilatation in the past and has been the gold standard for assessing labor progress [7, 8]. However, it can be subjective, inaccurate, and uncomfortable for women [9]. Also, only 50% of assessments are accurate, and there is an increased risk of infection with frequent DVE [8, 10]. Therefore, vaginal examination cannot be a correct scale for measuring cervical dilatation, mainly when done by different examiners [11]. Because of the poor reliability and pain associated with DVE, the use of intrapartum ultrasound to measure cervical dilation has been suggested as an alternative method [12]. Abdominal, vaginal, trans-labial, and trans-perineal 2-dimensional (2D) and 3D ultrasounds have been used to measure cervical dilatation during labor [13]. The first usage of trans-perineal ultrasonography for measuring the cervical os at various stages of labor was reported in 1996 by Voskresinsky [14]. In 2009, Zimerman et al. compared 3D ultrasound assessments of cervical dilatation and clinical examinations and found a significant correlation [15]. Hassan et al. found excellent agreement between trans-perineal ultrasound (TPUS) and DVE in measuring cervical dilation [11]. Trans-labial 3D ultrasonography has been suggested as an accurate and reproducible method for assessing cervical dilatation [15]. As a result, evidence indicates that the most frequently used imaging technology for measuring cervical dilatation is ultrasound [16]. However, which particular ultrasound modality is superior to others is unclear. It is also not clear how reliable ultrasound measurements are compared to vaginal examination. The current review aims of the current review is to verify the reliability of the ultrasound method compared with DVE in detecting cervical dilation.

## Materials and methods

This systematic review followed the methodology consistent with Systematic Reviews and Meta-Analyses of diagnostic testing studies. The protocol of this systematic review was published in 2019 [17].

### Search strategy

In this systematic review, we included promulgated studies until April 19, 2019. The search was updated on May 17, 2020. Frequent searches were performed on Cochrane Central Register of Controlled Trials (CENTRAL) via Cochrane Library, MEDLINE via Ovid, EMBASE via Ovid, CINAHL, ISI Web of Science Core Collection, Trip Database, PubMed Systematic Reviews subset, DARE, and NHS EED via the University of York, HTA and PROSPERO via the University of York (all databases were searched from inception to the current data). Ultrasound Methods, Cervical Dilatation, and Labor were used as key-words.

### Inclusion and exclusion criteria

**Fig. 1:**
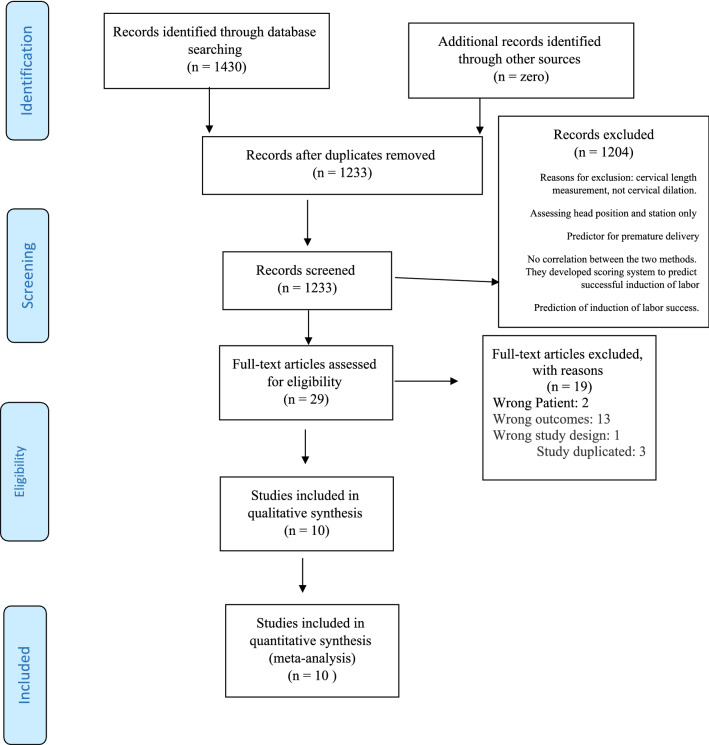
Flow diagram of the study

Included in this review were all observational studies with cross-sectional or diagnostic case–control study designs evaluating the accuracy of available methods for cervical dilation measurement during labor. We assessed measurement method alone or in combination with DVE (when used in a diagnostic algorithm). We did not impose any language restriction in this review. Studies that had the following characteristics were included: studies using ultrasound and DVE for detecting cervical dilation, and those which recruiting women with singleton pregnancy, with any type of placenta attachment or type of conception, at any maternal age, and body mass index. Studies that recruited pregnant women with twins, triplets, or quadruplets were excluded. All cited studies must have obtained informed consent from each and every study participant and received protocol approval from an ethics committee or institutional review board (Fig. 1).

### Study selection and data extraction

The search was carried out by MA. Two authors (MA and ZM) independently screened all searched studies and extracted data using Excel from those included in the review. If there was a conflict, it was resolved by discussion or getting advice from a third party (PA or SJ).

**Fig. 2:**
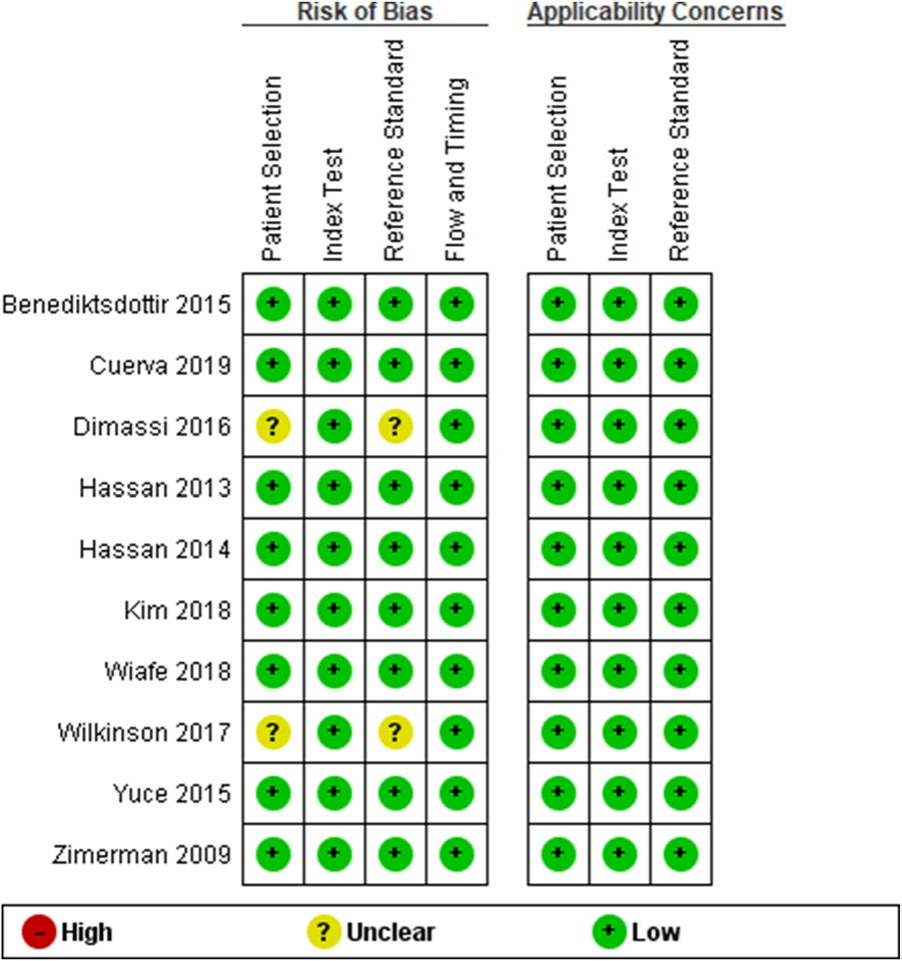
Risk of bias and applicability concerns summary: review authors’ judgments about each domain for each included study

We used the Quality Assessment of Diagnostic Test Accuracy Studies-2 (QUADAS-2) to assess the methodological quality of the included studies. The QUADAS-2 tool was applied in four phases: it summarizes the review question, tailors the instrument, and produces review-specific and judge bias and applicability. Each paper was judged as having a ‘low’, ‘high’, or ‘unclear’ risk for each of the four domains, and concerns about applicability were assessed in three disciplines. Two review authors (MA and ZM) independently applied the QUADAS-2 tool to the full text of each study. Disagreements were resolved by discussion, or if needed, by a third review author (SJ or PA). RevMan software was also used to construct methodological quality summary graphs. The summary of risk of bias assessment is presented in Fig. 2.

### Statistical analysis

The unit of analysis in studies was women in labor, as cervical dilatation is a single calculated measure using various methods. We extracted the intraclass correlation coefficient (ICC), and the Pearson correlation coefficient and *P* values associated with these measures to estimate the reliability of ultrasound compared with DVE. The data were then transferred into the Comprehensive Meta-Analysis Software to produce plots and estimates. We presented individual study results graphically by plotting the estimates of reliability in forest plots.

To facilitate comparisons across studies, eligibility was restricted to studies measuring reliability via: (1) intraclass correlation coefficient (ICC); (2) Pearson correlation coefficient, and (3) minimal detectable change with a 95% confidence level. The ICC, inter- and intra-tester reliability measure, essentially assess absolute agreement in repeated measurements of an object. ICC has been commonly used in the functional connectivity literature to assess reliability by some authors [18, 19]. Pearson’s correlation is used where variables are scaled and centered separately. It is used to assess the strength of a linear relationship between the results of two tests [20]. To maximize the number of studies included in the forest plot, when an article only reported the minimal detectable change with a 95% confidence level, it was transformed into SEM using the following formula:

1 $${\text{SEM}} = \frac{{{\text{MDC}}}}{1.96 \times \sqrt 2 }.$$

Then the SEM was converted into ICC using Formula (2).

Formula:

2 $${\text{ICC}} = \sqrt 1 - \frac{{{\text{SEM}}^2}}{{{\text{SD}}^2}}.$$

Pearson correlation coefficient was also converted to ICC, and the final meta-analysis was conducted using ICC values. Using raw ICC values, we analyzed the data with the assumption that they were distributed normally. To perform a meta-analysis of pooled ICC using the random-effects model, all coefficients were transformed to Fisher’s *Z* values and weighted by sample size using inverse variance weight for the analysis. The average reliability coefficients and their confidence limits were back-transformed to the original metric of reliability coefficients to facilitate the interpretation of the results. ICC values of 0.7–0.9 were considered acceptable, but values higher than 0.9 deemed desirable [21].

The heterogeneity was assessed by calculating the *Q* statistic and the *I*^2^ index. The *I*^2^ above 50% was considered heterogeneous. The statistical analyses were carried out with Comprehensive Meta-analysis 3.3 software (BioStat, Englewood, NJ, USA) [22]. Additionally, a funnel plot was drawn to estimate the publication bias.

## Results

In the primary search of databases, 1430 articles were found. After removing the duplicates (*n* = 1233), the titles and abstracts were screened for potentially relevant studies (*n* = 1222). Eleven articles were considered eligible for full-text screening. We attempted to contact the authors to obtain the complete text for one article, but received no reply, so we excluded this study [23]. Finally, ten studies were included in the meta-analysis (Fig. 1).

The included studies were published from 2009 to 2019 with a prospective cohort design. The characteristics of the included studies are shown in Table 1. Three of the studies were conducted in the UK [11, 12, 24], and seven in each of following countries: Spain [25], South Korea [7], Republic of Ghana [3], Tunisia [26], Sweden [27], Turkey [28], and Israel [15]. All studies were performed in the hospital setting. TPUS or trans-labial ultrasound (TIUS) versus DVE was used for the assessment of cervical dilatation during labor in all studies. The transducer of the ultrasound examinations was placed transperineally at the level of the posterior Fourchette in a sagittal position. Vaginal digital examinations were performed before or immediately after the ultrasound examination by the responsible birth attendant. Six studies recruited both nulliparous and multigravida women [3, 7, 15, 24, 25, 28], and one study enrolled only nulliparous women [11]. One study used TLUS (trans-labial ultrasound) method, and other studies adopted TPUS (trans-perineal ultrasound) method. The sample size of all studies ranged from 25 to 195. The total number of participants enrolled was 856 in both groups.

**Table 1:** Characteristics of studies included in the systematic review

Study	Location	Study type	Sample size	Parity			Age median (range)/mean ± SD	Gestational age (weeks): median (range)/mean ± SD	BMI: kg/m^2^ median (range)/mean (SD)	Single or multiple	Presentation	Intervention	Control
				Nulliparous		Multi							
Cuerva et al. [25]	Spain	Prospective cohort	57	31.5%		68.5%	31.6 ± 6.3	39.2 ± 0.9	24.3 ± 5.4	Singleton	Cephalic	TPUS	DVE
Kim et al. [7]	South Korea	Prospective cohort	25	72%		28%	32 (18–38)	38.4 (32.8 -40.8)	–	Singleton	Cephalic	TPUS	DVE
Wiafe et al. [3]	Republic of Ghana	Prospective cohort	195	Nulli	Primi	31%	27 Mean	39 ± 4	28 (20–42)	Singleton	Cephalic	TPUS	DVE
				47%	22%								
Wilkinson et al. [24]	UK	Prospective cohort	195	93%		7%	31 (18–44)	40 (24–42)	24.1 (15.7–42.4)	Singleton	Cephalic	TPUS	DVE
Dimassi et al. [26]	Tunisia	Prospective cohort	60					Not mention	Not mention	Singleton	Cephalic	TPUS	DVE
Yuce et al. [28]	Turkey	Prospective cohort	43	58%		42%				Singleton	Cephalic	TPUS	DVE
Benediktsdotti et al. [27]	Sweden	Prospective cohort	86	Median (range): 1 (0–5)			30.5 (23–43)	40 (36–42)	24.3 (18–36)	Singleton	Cephalic	TPUS	DVE
Hassan et al. [12]	UK	Prospective cohort	52	Median(range): 0 (0–2)			34 (21–41)	40 (37–41)	29 (22–47)	Singleton	Cephalic	TPUS	DVE
Hassan et al. [13]	UK	Prospective cohort	40	100%		–	Not mentioned	Not mentioned	Not mention	Singleton	Cephalic	TPUS	DVE
Zimerman et al. [15]	Israel	Prospective cohort	52	43%		57%	29.8 ± 4.23	39.7 ± 1.32		Singleton	Cephalic	TLUS	DVE

*Primi* primigravida, *Multi* multigravida, *y* years, *MI* body mass index, *TPUS* trans-perineal ultrasound, *TLUS* trans-labial ultrasound, *DVE* digital vaginal examination

### Overall meta-analysis

**Fig. 3:**
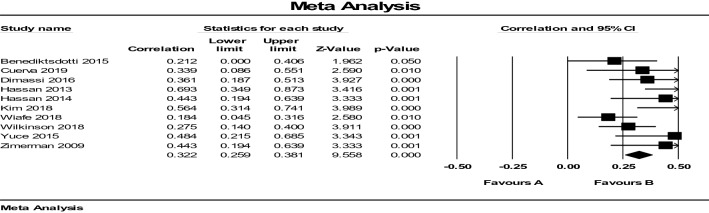
Average intra-tester and inter-session reliability of ICC between ultrasound measurements and digital examinations of cervical dilatation

We analyzed ten studies with a total sample size of 856. The ICC values ranged between 0.21 and 0.69. The fixed-effect models for the ultrasound test showed average reliability of ICC (*r* = 0.32 (95% CI 0.26–0.38). Heterogeneity was estimated using *I*^2^ = 48.72 (Table 2). The effect sizes exhibited moderate heterogeneity (based on the *Q* statistics and the *I*^2^ indices), supporting the decision to apply the random-effects model. Correlation between the two methods with random model was *r* = 0.359, (95% CI 0.26–0.44, *P* = 0.000). The limits of agreement were 0.267–0.446. Based on the value of the ICC with a 95% confidence interval, the correlation between the two methods for the measurement of cervical dilatation was poor. Forest plot of the intra-tester and inter-methods reliability as seen in Fig. 3 was obtained in the studies that applied TPUS or TIUS versus DVE to measure cervical dilatation.

**Table 2:** Effect size model for heterogeneity estimated

Model	Effect size and 95% interval				Test of null (2-tail)		Heterogeneity				Tau-squared			
Model	Number studies	Point estimate	Lower limit	Upper limit	*Z*-value	*P* value	*Q*-value	df (*Q*)	*P*-value	*I-*squared	Tau-squared	Standard error	Variance	Tau
Fixed	10	0.322	0.259	0.381	9.558	0.000	17.550	9	0.041	48.719	0.012	0.013	0.000	0.111
Random	10	0.359	0.267	0.446	7.157	0.000

### Sub-group analysis by gravidity

**Fig. 4:**
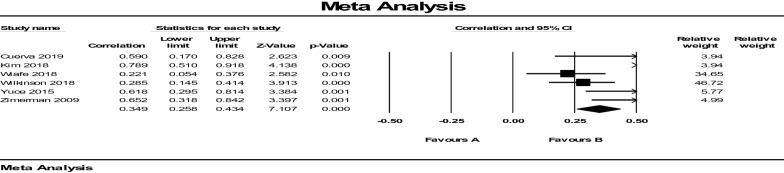
Average intra-tester and inter-session reliability of ICC between ultrasound measurements and digital examinations of cervical dilatation in nulliparous

**Fig. 5:**
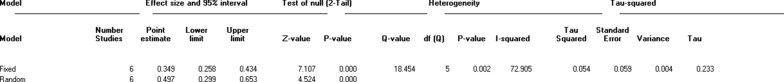
Effect size model for heterogeneity estimated in nulliparous

Seven studies included 439 nulliparous women, and six studies included 168 multigravida women. Overall, ICC reliability was 0.32 (95% CI 0.26–0.38). The fixed-effect model showed that in nulliparous participants the correlation between ultrasound measurements and digital examinations for measurement of cervical dilatation during labor is *r* = 0.349, (95% CI 0.25–0.43 *P* = 0.000) (Fig. 4). The limits of agreement were 0.258–0.434. The *I*^2^ was 72.905, which means that 72% of the observed variance between studies is due to fundamental differences in the effect size. Only about 28% of the observed variance would have been expected based on random error. Tau-squared is 0.054. This is the “between studies” variance that was used in computing weights. The random-effect model was then used to give more weight to smaller studies. The correlation between the two methods with the random-effect model is *r* = 0.497 (95% CI 0.29–0.65 *P* = 0.000) (Fig. 5). Based on the value of the ICC with 95% confident intervals, the correlation between the two methods was poor in the nulliparous (ICC lower than seven).

**Fig. 6:**
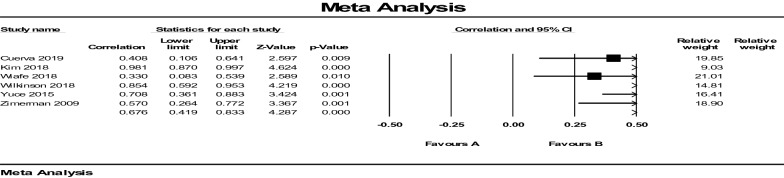
Average intra-tester and inter-session reliability of ICC between ultrasound measurements and digital examinations of cervical dilatation in multigravida

**Fig. 7:**
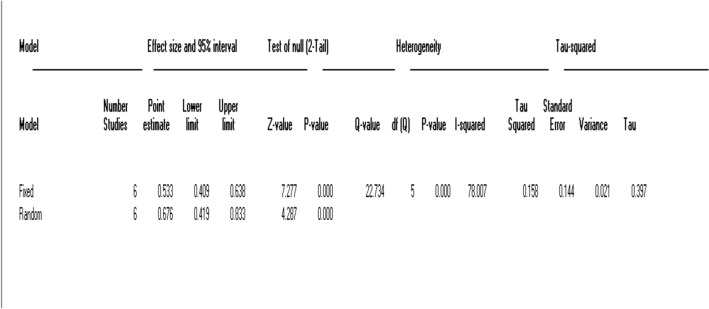
Effect size model for heterogeneity estimated in multigravida

In multigravida women, as seen in Fig. 6, because Tau-squared is 0.158, the random-effect models for the ultrasound test were used, and results showed an average value [ICC (*r* = 0.676, 95% CI 0.419–0.833), *P* = 0.000]. In this model, *I*^2^ was 78.007, showing the correlation between the two methods is moderate in the multigravida women (Fig. 7).

## Discussion

### Summary of main findings

The primary purpose of the current meta-analysis was to estimate both the inter- and intra-methods reliability of using ultrasound compared to DVE in detecting cervical dilation measures. In this systematic review, ten studies were included. Overall, the random model showed poor reliability between the two methods. This can be because the women participating in these studies were not homogenous in terms of parity.

The subgroup analysis showed that the correlation between DVE and ultrasound in nulliparous women was poor, while this correlation in multiparous women was moderate. This means that in multigravida women, ultrasound measurements and digital examinations for cervical measures during labor are consistent. Overall, the pooled data indicated a low value of ultrasound diagnosis, resulting in conflicts with independent studies.

DVE is still the most commonly utilized method to assess cervical dilatation, fetal presentation, fetal position, and fetal descent during all stages of labor. However, DVE is associated with pain and the risk of infection. Therefore, clinicians tried to replace DVE with other methods, such as trans-perineal ultrasound.

The texture of the cervix changes dramatically after the first birth. Some women undergo rupture of the cervix at birth. Therefore, it is logical that multipara women would have a differently shaped cervix compared with their nulliparous counterparts [29]. It is also possible that the cervix drastically remodels, reorganizes, and softens during gestation. Thus, the consistency and integrity of the cervix vary at different gestational age. As the fetus descends to the pelvis, more pressure is placed on the cervix. Hence, the length of the cervix is expected to shorten as a pregnancy progresses [30].

This is especially true in nulliparous women as the fetal descent happens during the last 4 weeks of pregnancy, and it is a slow descent, rather than a fast one, as seen in multiparous pregnancies. Moreover, women with elongated cervix might have more fiber in the cervix, making the cervix’s mechanics and structure different from those with the shorter cervixes [31].

In this study, we found a low value of ultrasound diagnosis, which conflicts with the independent studies. This could be due to the high heterogeneity found in our pooled data. The resolution lies in more sample size, which translates to conduct more quality RCTs. Furthermore, some of the included studies had very low sample sizes, and the effect of confounders such as the timing of membrane rupture, was not apparent. The studies also failed to mention whether the data were collected during the active or latent phase of labor.

The preliminary results of Zimerman et al. showed that ultrasound to detect cervical dilation is considered problematic [15]. However, Hassan et al. showed that the correlation coefficient between ultrasound measurements and DVE is relatively high (*r* = 0.82, *P* = 0.05) [11, 12].

Also, Wiafe et al. in a systematic review showed a high correlation between ultrasound and digital examination of the cervix for detecting cervical dilation. Still, there was no significant difference in terms of success rate [32]. The discrepancy between the present study and the Wiafe et al.’s study may be related to the fact that they recruited five studies. The heterogeneity in their meta-analysis was high (*I*^2^ = 96%), and they did not follow the DTA method.

DVE is the accepted clinical procedure for the detection of cervical dilatation during labor [33]. However, DVE is a manual procedure that heavily depends on the providers’ experience. It is therefore, considered an imprecise measurement if conducted by inexperienced clinicians [34]. In addition, examination and manipulation of the cervix might cause discomfort to women. In contrast, in ultra-sonographic cervical dilatation measurement, the uterine cervix is left intact, and natural contour is preserved [15]. Also, cervical dilation changes in labor according to studies that used cervical ultrasound markers (clips) over time. Thus, two examiners may differ and yet both might be accurate [35]. Martorelli et al. also concluded that transvaginal ultrasound before the onset of labor in women with gestational age > 40 weeks might help predict failed labor induction. Still, it should not be used for performing a cesarean section [36].

### Strengths and limitations

This was the first systematic review to compare the reliability of ultrasound (TPUS or TLUS versus digital examination in detecting cervical dilation. The quality of the included studies was good, and most studies were free of serious biases.

Several limitations existed in this meta-analysis: (1) three studies failed to report parity; hence we were unable to include these studies in our subgroup analysis; (2) some other confounders such as the timing of rupture of member and the active or passive phases of labor were not evident; and (3) the sample size of the included studies was very small. These limitations could have contributed to heterogeneity substantially.

### Clinical application

According to this systematic review, the digital examination can be replaced by trans-perineal ultrasound in multiparous women, while using this method in nulliparous women needs more thorough studies.

## Conclusion

Trans-perineal ultrasonography seems to be a reliable method for assessing labor progression in multigravida women, but its application in nulliparous women needs further studies.

## Data Availability

Not applicable.

## Abbreviations

ICC: Intraclass correlation coefficient
VE: Vaginal examination
DVE: Digital vaginal examination
QUADAS-2: Quality Assessment of Diagnostic Test Accuracy Studies-2
TLUS: Trans-labial ultrasound
TPUS: Trans-perineal ultrasound method
